# Molecular Interaction of Protein-Pigment C-Phycocyanin with Bovine Serum Albumin in a Gomphosis Structure Inhibiting Amyloid Formation

**DOI:** 10.3390/ijms21218207

**Published:** 2020-11-02

**Authors:** Yi-Cong Luo, Pu Jing

**Affiliations:** Shanghai Food Safety and Engineering Technology Research Center, Bor S. Luh Food Safety Research Center, Key Lab of Urban Agriculture (South), School of Agriculture & Biology, Shanghai Jiao Tong University, Shanghai 200240, China; luoyicong0021@sjtu.edu.cn

**Keywords:** C-phycocyanin, blue food pigment, secondary interaction, fibrillation, non-covalent interaction

## Abstract

Accumulation of amyloid fibrils in organisms accompanies many diseases. Natural extracts offer an alternative strategy to control the process with potentially fewer side effects. In this study, the inhibition of C-phycocyanin from *Spirulina sp.* on amyloid formation of bovine serum albumin (BSA) during a 21-day incubation was investigated using fluorescence and circular dichroism (CD), and mechanisms were explored via kinetic fitting and molecular docking. C-phycocyanin (0–50 µg/mL) hindered the amyloid formation process of BSA with increased half-lives (12.43–17.73 days) based on fluorescence intensity. A kinetic model was built and showed that the k_1_ decreased from 1.820 × 10^−2^ d^−1^ to 2.62 × 10^−3^ d^−1^ with the increase of C-phycocyanin, while k_2_ showed no changes, indicating that the inhibition of BSA fibrillation by C-phycocyanin occurred in a spontaneous process instead of self-catalyzed one. CD results show that C-phycocyanin inhibited conformational conversion (α-helices and β-sheets) of BSA from day 6 to day 18. Molecular docking suggested that C-phycocyanin may hinder BSA fibrillation by hydrogen-bonding > 6 of 27 α-helices of BSA in a gomphosis-like structure, but the unblocked BSA α-helices might follow the self-catalytic process subsequently.

## 1. Introduction

The process of aggregation and fibril formation called amyloid formation is widely found in proteins of organisms with unknown physiological functions. Accumulation of amyloid fibrils in organisms accompanies many diseases, such as Alzheimer’s, Parkinson’s, diabetes, prion diseases, etc. [1,2]. The mechanism of amyloid formation is still debated. Many different precursor proteins are involved in the fibrillation process; protein fibrillation is often accompanied by a decrease of α-helices and an increase of β-sheets [3]. Amyloid deposits have been under investigation in a number of pathological states, which then cause irreversible damage to the tissue [4], such as amyloid-β(Aβ) plaque deposits with neurofibrillary tangles containing tau proteins, which are the key pathognomonic manifestations of Alzheimer’s disease [5].

A range of small molecules (e.g., peptides and polyphenols) have been identified as impeding fibril formation. Peptides inhibitors or monomer binding-protein have been designed to block self-association and protein aggregation as a potential therapeutic target [6,7], although numerous phytochemicals such as polyphenols have been investigated to remodel mature amyloid fibrils to non-toxic aggregates [8,9]. A previous study showed that oral administration of the cyanobacteria *Spriulina* appeared to mediate effects. *Spirulina* exhibited a neuroprotective role against 1-methyl-4-phenyl-1,2,3,6-tetrahydropyridine neurotoxicity in a mice model of Parkinson’s disease. *Spirulina* may prevent memory dysfunction, possibly by lessening Aβ protein accumulation, reducing oxidative damage and mainly augmenting the catalase activity in senescence-accelerated mice [10]. The pigment compound, namely C-phycocyanin, has been implicated in protective benefits [11,12]. C-phycocyanin affected astrocytes-mediated neuroprotection against oxidative brain injury in astrocyte tissue models [11]. More recently, C-phycocyanin from *Spirulina* was found to inhibit α-synuclein and amyloid-β fibril formation [13]. Molecular docking studies [14] showed that the phycocyanin interacted with the enzyme β-secretase, which catalyzes the proteolysis of the amyloid precursor protein to form plaques.

**Figure 1:**
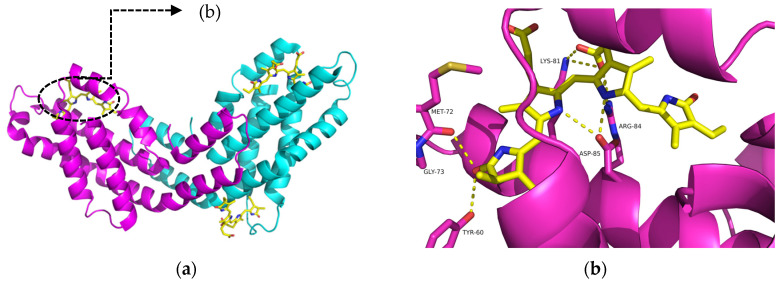
Structure of C-phycocyanin (**a**) and phycocyanobilin (yellow) with its secondary interaction of amino acid residues on the α subunit (**b**). The α and β subunits are colored in pink and blue, respectively.

C-phycocyanin is a homologous dimer containing two identical peptides and three chromophores, and is practically recognized as a monomer, which can self-assemble into trimer or hexamer in a solution based on concentration [15]. C-phycocyanin (Figure 1a) from *Spirulina sp.* is an edible pigment protein, composing of α and β subunits. Three tetrapyrrole chromophore phycocyanobilins (Figure 1b) are covalently attached to the αβ monomer through three conserved cysteine residues [16]. Additionally, a variety of secondary interactions occur between phycocyanobilin and chromopeptide.

Bovine serum albumin (BSA) is a helical protein containing 583 amino acids, and is the most abundant protein in the circulatory system that is involved in the transport of drug molecules. BSA can spontaneously form fibers at pH = 7.4, with the increase of β-sheets [17]. Therefore, BSA is often used as a model of protein fibrillation [18,19,20]. In this study, we aimed to investigate the inhibition of C-phycocyanin from *Spirulina sp.* on the fibrillation of BSA. This was evaluated using fluorescence and circular dichroism analyses and to study the mechanism of the secondary interaction using molecular docking.

## 2. Results

### 2.1. Inhibitory Effect of C-phycocyanin on Formation of Amyloid Fibrils

Fluorometric and CD assays were applied to evaluate the process of amyloid formation [21,22]. The precipitates of insoluble protein fiber started being visually notable until day 18 and plenty were generated on day 21.

### 2.2. Fluorescence Intensity and Kinetic Fitting Results

**Figure 2:**
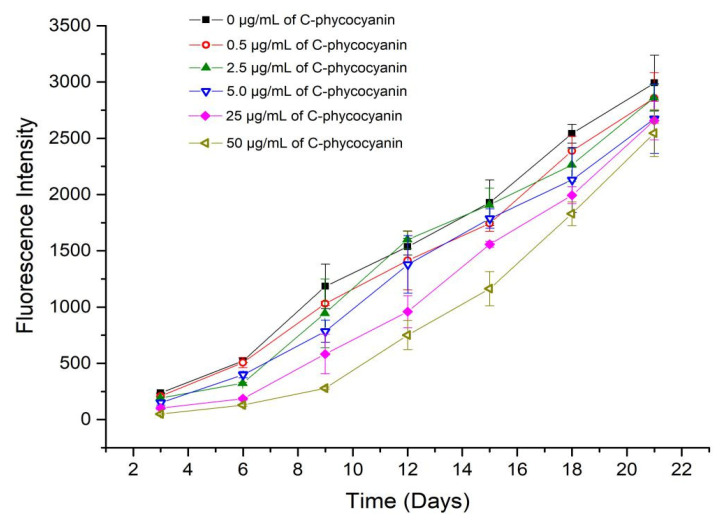
Inhibitory effect of C-phycocyanin on amyloid formation using fluorometric assay for a 21-day duration.

The fluorescence intensity was positively associated with protein amyloid formation due to the characteristics of fluorescence, and showed in the linear range at low concentrations of BSA <~260 µM [23], where 5 µM of BSA was applied in the experiment. Figure 2 shows that the fluorescence intensity as an index of the degree of inhibitory effect on the amyloid formation of BSA was negatively associated with an increasing dosage of C-phycocyanin (0.5–50 µg/mL), suggesting a potential interaction between BSA and C-phycocyanin. Considering these measurements contained time as a variable, repeated measures analysis of variance was selected for statistical analysis. The statistical data with the same dosage of C-phycocyanin were defined as a group. Results of intergroup effect analysis suggested the effect of different dosages of C-phycocyanin on the amyloid formation of BSA fibrosis was significantly different (*F* = 11.24, *p* = 0.0003 < 0.001). In the meantime, due to the result of sphericity test being 5.72 × 10^−8^ < 0.05, Greenhouse–Geisser correction was employed. Multivariate analysis of variance revealed an extremely significant main effect of time (*F* = 1496.4, *p* = 5.691 × 10^−30^ < 0.001, Greenhouse–Geisser corrected). However, the fluorescence intensity in each treatment gradually increased during a 21-day incubation indicated that C-phycocyanin was unable to completely terminate the transformation from BSA to amyloid.

Table 1 shows the kinetic fitting results of fluorescence analysis, whereas the kinetic fitting plots (Figures S1–S6) are presented in the Supplementary Materials. All ‘m’ values (3.6 × 10^3^) in treatment groups (0.5–50 µg/mL) were fixed so that the results could definitely be comparable. The coefficients of k_1_ and k_2_ were designed for two types of amyloid formation: k_1_ represented the process that BSA forms amyloid spontaneously, whereas k_2_ was for the self-catalyzed amyloid formation. The half-life (t_1/2_) values of amyloid formation were calculated and increased obviously from 12.43–17.73 days with the concentration of C-phycocyanin from 0–50 µg/mL, indicating that C-phycocyanin exhibited remarkable inhibitory effect. The variation of k_1_ (~10^−2^ to 10^−3^) was significant compared to k_2_ (~10^−5^), suggesting that k_1_ was the prominent coefficient of the kinetics in the inhibitory process of C-phycocyanin on amyloid formation.

### 2.3. CD Spectra

**Figure 3:**
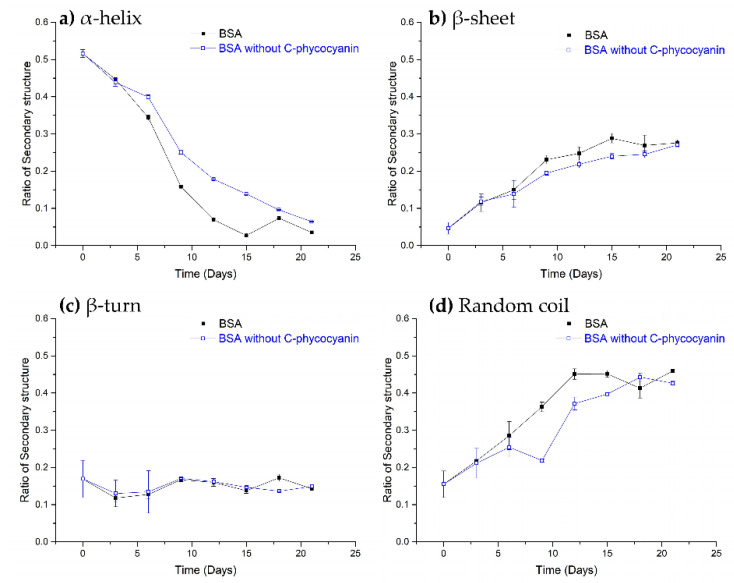
Variation on the secondary structure ((**a**), α-helix; (**b**), β-sheet; (**c**), β-Turn; (**d**), random coil) of BSA complexes with C-phycocyanin (50 µg/mL) calculated using the CDSSTR software during a 21-day incubation.

CD spectra were analyzed and the ratios of secondary structures were calculated to determine the process of BSA amyloid formation in Figure 3 according to the references [3,24]. Figure 3a,b showed that BSA alone changed by a decrease of α-helices and an increase of β-sheets during a 21-day incubation, which was noted as a typical structural change in fibrillation formation [25]. Similar statistical analysis as above was selected for the difference of α-helix, β-sheet and β-Turn between two groups. The insignificant variation of the secondary structures including ‘β-Turn’ (*F* = 0.002, *p* = 0.964 > 0.05) was observed during the incubation in Figure 3c, indicating that it might not be involved in fibrillation formation. The formation of ‘Random coil’ increased with days, suggesting that it may play an important role in amyloid formation. Results of intergroup effect analysis suggested that the effect of C-phycocyanin on the amyloid formation of BSA fibrosis was significantly different (F_α-helix_ = 1208.18, *p*_α-helix_ = 4.088 × 10^−6^ < 0.001 and F_β-sheet_ = 17.66, *p*_β-sheet_ = 0.014 < 0.05). Thus, the addition of C-phycocyanin in Figure 3a,b diminished the decrease of α-helices and the increase of β-sheets in BSA from day 6 to day 18, compared to the BSA alone, suggesting that C-phycocyanin interrupted the transformation and finally prolonged the amyloid formation of BSA. Meanwhile, C-phycocyanin notably affected the formation of ‘Random coil’ in Figure 3d.

### 2.4. Molecular Docking

**Figure 4:**
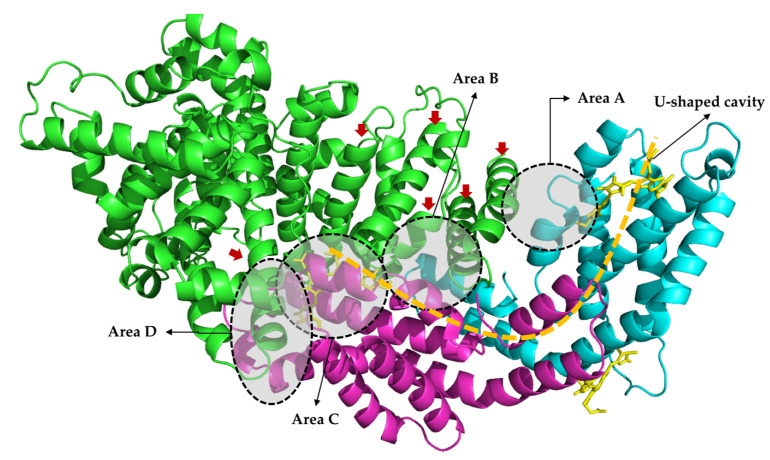
Interaction of BSA (PDB id: 4f5s) and C-phycocyanin (PDB id: 2vjr) using molecular docking. Solvent molecules were removed before molecular docking. The green chain is BSA. α and β subunits of C-phycocyanin are colored in pink and blue, respectively, whereas phycocyanobilin is yellow. Red arrows represent the BSA α-helices blocked by C-phycocyanin.

The C-phycocyanin molecules practically recognized as a monomer (PDB id: 2vjr) containing two non-separable homologous units, and a self-assembled trimer or hexamer (PDB id: 1phn) in water [15] were first considered to dock with BSA (PDB id: 4f5s) in this study. The monomer showed much more interaction with BSA than others and was finally selected as the C-phycocyanin molecular module for docking analysis. The optimized molecular docking model is shown in Figure 4, where C-phycocyanin interacted with BSA by binding to ~6 of 27 α-helices in BSA in a U-shaped cavity, likely a type of gomphosis structure where C-phycocyanin from bottom fixed BSA.

**Figure 5:**
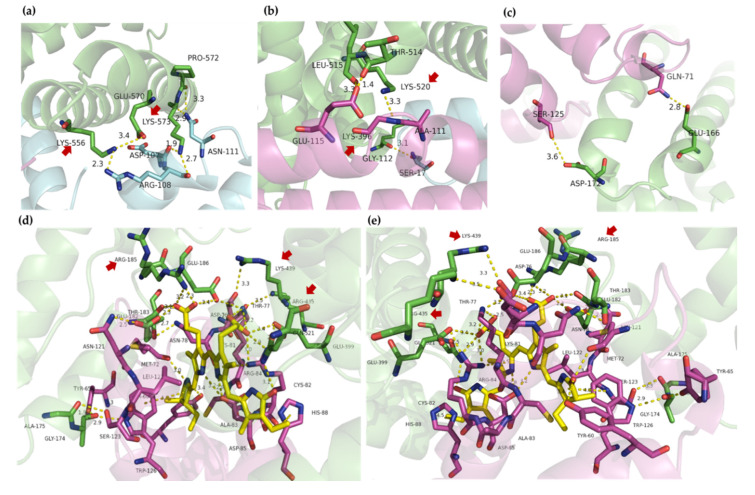
Secondary interaction of areas A (**a**), B (**b**), D (**c**) and C (**d**,**e**) in docking models. The green chain is BSA, whereas the pink and blue ones are α and β subunits of C-phycocyanin, respectively. Area C containing phycocyanobilin (yellow structure) is viewed from different angles (**d**,**e**). Red atoms are oxygen and blue atoms are nitrogen. Hydrogen bonds are shown in yellow dotted lines. Unit of length is Å.

**Figure 6:**
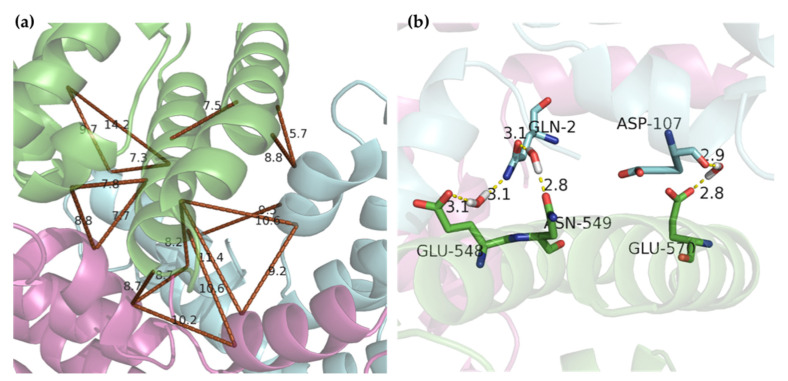
Hydrophilic cavity (**a**) in areas A and B and a possible example in area A (**b**). Some distances were measured and are shown in brown lines. The example is in the triangle with side lengths of 11.4, 9.2 and 10.6 Å. The gray atoms are hydrogen atoms in the left panel. The green chain is BSA. The pink and blue chains are α and β subunits of C-phycocyanin, respectively.

Four characteristic areas, A-D, in Figure 4 were defined in order to explore the secondary interactions. In these areas, a large amount of hydrogen bonds was found and most hydrogen bonds possessed a bond length of 2.0–3.5 Å. Some groups, like peptide linkage and –NH_2_ or –NH_3_^+^ on the chain side of amino acid residues, were good hydrogen bond donors, while carbonyl and carboxyl may perform as good hydrogen bond acceptors. The α subunit of C-phycocyanin played a key role with numerous X-H⋅⋅⋅Y and X-H⋅⋅⋅π hydrogen bonds, suggesting the significance of cofactors in the docking model. Figure 5a shows that Lys-556 and Lys-573 of BSA in area A were blocked by the residues from the β subunit of C-phycocyanin via hydrogen bonding. Similarly, Lys-520 and Lys-396 in area B were blocked in Figure 5b. The hydrogen bonds shown in areas A and B contributed to the increase in the boundary area between BSA and C-phycocyanin, and then allowed two α-helices from BSA to enter into the U-shaped cavity of the C-phycocyanin vertically. Additionally, many hydrophilic residues (53.4% in total) were 25, 21 and 16 residues from BSA, α and β subunits in the cavity, respectively, and contributed to the hydrophilicity of the cavity. Distances among helices in the cavity were about 7–11 Å in Figure 6a. As a result of hydrophilicity and spaces of the cavity, water (diameter: ~ 4 Å) was able to fill in and potential was able to build up cross-bridge linkages among polypeptides via hydrogen bonding (Figure 6b), so as to access extra solvation energy.

Almost all of tested models contained similar interactions shown in areas C and D, indicating that they were the core part of the docking models and provided the most stabilizing energy. Area C in Figure 5d,e shows mainly secondary interactions within the α subunit and phycocyanobilin of C-phycocyanin. The Arg-185, Lys-439, and Arg-435 from BSA were blocked by C-phycocyanin via hydrogen bonding.

## 3. Discussion

A kinetic model was established to study the spontaneous and self-catalytic process in BSA fibrillation, as well as to evaluate the inhibitory effect on amyloid formation by C-phycocyanin. The model was simplified with a hypothesis that the BSA amyloid is a monomer and does not exhibit multiple molecular action, since numerous dimer–aggregate and aggregate–aggregate interactions occurred in differential equations, resulting in unlikely solved multiple variables [26]. However, all factors including the spontaneous transformation from BSA to amyloid [27], the polymerization-dependent induction of amyloid to the process of BSA fibrillation [28], the mutual aggregation of polymerization-dependent amyloids [29] and the polymer solubility [30] may need to be considered comprehensively for an accurate kinetic model in further studies.

C-phycocyanin (0~50 µg/mL) hindered the amyloid formation process of BSA with increased half-lives (12.43–17.73 days) based on fluorescence intensity and affected spontaneous process (k_1_), but not the self-catalysis process (k_2_) based on the kinetic model. CD spectra showed that C-phycocyanin inhibited conformational conversion of BSA by decreasing α-helices and increasing β-sheets from day 6 to day 18, which was consistent with the fluorescence study.

**Figure 7:**
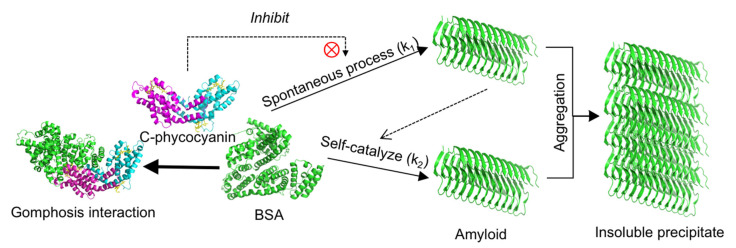
Mechanisms of phycocyanin inhibition on amyloid formation.

Molecule docking analyses showed that C-phycocyanin appeared to interact with one side of BSA in a type of gomphosis structure, and differed from most of the small inhibitors that combined and stabilized BSA from the inside [31,32]. Since α-helix was the essential structure for spontaneous transformation in amyloid formation [33], C-phycocyanin might hinder the process of fibrillation, likely by decelerating the spontaneous transformation in amyloid formation. However, BSA still had a large number of free α-helices and followed the spontaneous process to form amyloids, which might catalyze others into fibrillation in the self-catalytic process (k_2_) in the kinetic model. That may explain why k_2_ was little affected by C-phycocyanin. In other words, k_1_ declined because some α-helices were blocked, whereas k_2_ showed no significant changes because the free α-helices might be the major part involved in the self-catalytic process. The mechanism of C-phycocyanin inhibiting amyloid formation was schemed in Figure 7.

Hydrogen bonds in areas A and B allowed two α-helices from BSA to enter into a U-shaped cavity of the C-phycocyanin vertically. Areas C and D, as the core part of the docking models, provided the most stabilizing energy. The mechanism of the inhibition of C-phycocyanin on amyloid formation is likely to inhibit the spontaneous transformation step (k_1_) of BSA by stabilizing at least 6 α-helices, whereas k_2_ showed no significant changes because the unblocked α-helices might exactly be the major part involved in the self-catalytic process. Additionally, alkaline amino acids, i.e., lysine and arginine, can participate in a glycation reaction [34], which plays the role in stimulating amyloid aggregation [35]. As a result of the docking model, around 37.5% lysines and 47.6% arginines of BSA have been blocked, and thus potentially rendered the glycation process, which is consistent with the previous study of quercetin on BSA glycation [36].

## 4. Materials and Methods

### 4.1. Materials

C-phycocyanin (from *Spirulina sp.*) was purchased from Sigma-Aldrich (Shanghai, China) with a purity of 99% determined by HPLC. Albumin from Bovine Serum (BSA) with a purity of 96% and ThT (thioflavine T) were purchased from Aladdin Bio-Chem Technology (Shanghai, China). All other chemicals used were of analytical reagent grade.

### 4.2. Inhibitory Effect of BSA on Amyloid Formation In Vitro

The inhibitory assay was designed according to the previous method [37] using Tecan Infinite M200 Pro microplate reader (Männedorf, Switzerland). The final volume of the reaction system was 1000 µL, containing BSA (0.1 mM, 50 µL) and various volumes of C-phycocyanin (1 mg/mL). The solvent was phosphate buffer (0.1 mM, pH 7.4). Samples were incubated in Eppendorf tubes at 25 °C for 21 days and were detected by fluorometric assay with ThT (0.02 mM, 100 µL) according to previous research [38]. ThT is able to combine with β-sheets to produce strong fluorescence (λex = 440 nm, λem = 484 nm) [39]. The interference of C-phycocyanin on fluorescence has been eliminated; in fact, no fluorescence was detected for the C-phycocyanin at λex = 440 nm. A system including ThT (0.02 mM, 100 µL) with phosphate buffer (0.1 mM, pH 7.4, 900 µL) was used to eliminate the interference of the solvent.

### 4.3. Kinetic Analysis

A kinetic model was established to study the spontaneous and self-catalytic process in BSA fibrillation, as well as to evaluate the inhibitory effect on amyloid formation by C-phycocyanin. In this kinetic model, A represents BSA and B represents amyloid. The kinetic constant of the spontaneous process is k_1_, and the kinetic constant of the self-catalytic process is k_2_.

$$A\overset{k_{1}}{\to }B$$

$$A+B\overset{k_{2}}{\to }2B$$

A differential equation was presented via this model.

(1) $$\frac{d\left[B\right]}{\mathrm{dt}}=k_{1}\left[A\right]+k_{2}\left[A\right]\left[B\right]$$

With a hypothesis that the total amount of BSA and amyloid is constant ([A] + [B] = m), the differential equation was calculated and then came to an implicit expression.

(2) $$t=\frac{1}{k_{1}+{\mathrm{mk}}_{2}}\ln\left(\frac{k_{2}\left[B\right]+k_{1}}{m-\left[B\right]}\right)-\frac{1}{k_{1}+{\mathrm{mk}}_{2}}\ln\left(\frac{k_{1}}{m}\right)$$

### 4.4. Circular Dichroism Analysis

Far-UV CD spectra (190–260 nm) were recorded on a JASCO J-815 spectropolarimeter (Jasco Inc., Easton, MD, USA). The scan speed was 100 nm/min with 1 nm bandwidth. The BSA (0.1 mM, 50 µL) alone or together with C-phycocyanin (1 mg/mL, 10 µL) was measured using a quartz cell of 1 mm path length. For the best signal to noise ratio (SNR), each absorbance curve was smoothed automatically. Base lining and analysis were done using Jasco J-720 software. In addition, CD spectra of C-phycocyanin in experimental condition were determined, yet there was no signal due to low concentration of C-phycocyanin in this study.

### 4.5. Molecular Docking

Z-Dock Server 3.0.2 [40,41] and its score function [42] were used for molecular docking. Protein preparation was carried out by adding hydrogens, removing water molecules. All possible binding patterns between two proteins via translation and rotation were studied, and used an energy-based scoring function to evaluate each potential pattern. The crystal structure in the protein data bank was applied as monomer (PDB id: 2vjr), trimer (PDB id: 1phn) and hexamer (PDB id: 1phn) of C-phycocyanin for molecular docking with BSA (PDB id: 4f5s). The docking data were rendered and screened using PyMOL Viewer [43].

### 4.6. Data Analysis

All the experiments were conducted in triplicate. CD data were analyzed using CDpro software to characterize the variation of the secondary structure of BSA during amyloid formation. Three algorithms including SELCON3, CONTIN/LL and CDSSTR were compared [24]. Among them, CDSSTR was selected for better results while SP-37A (SOLUBLE) was used as the reference protein. Curve fitting for the kinetic model was done using Origin 2017 64-bit software. Data are expressed as the mean ± standard deviation.

## Figures and Tables

**Table 1:** Kinetic fitting result of amyloid formation.

C-phycocyanin (µg/mL)	k_1_	k_2_	R^2^	Half-Life (t_1/2_, Days)
0.0	1.820 × 10^−2^ ± 3.14 × 10^−3^	4.198 × 10^−5^ ± 2.68 × 10^−5^	0.9931	12.43
0.5	1.445 × 10^−2^ ± 2.07 × 10^−3^	4.003 × 10^−5^ ± 4.28 × 10^−6^	0.9945	14.41
2.5	1.256 × 10^−2^ ± 3.42 × 10^−3^	4.894 × 10^−5^ ± 8.83 × 10^−6^	0.9804	13.89
5.0	1.247 × 10^−2^ ± 1.77 × 10^−4^	4.444 × 10^−5^ ± 4.63 × 10^−6^	0.9939	14.69
25.0	5.610 × 10^−3^ ± 9.42 × 10^−4^	5.930 × 10^−5^ ± 5.04 × 10^−6^	0.9928	16.45
50.0	2.620 × 10^−3^ ± 3.03 × 10^−4^	7.012 × 10^−5^ ± 3.34 × 10^−6^	0.9969	17.73

## Supplementary Materials

The following are available online at https://www.mdpi.com/1422-0067/21/21/8207/s1. Figure S1: CD data of BSA without the existence of C-phycocyanin; Figure S2: CD data of BSA with the existence of C-phycocyanin.; Figure S3: Two models between BSA and monomer of C-phycocyanin with less interactions; Figure S4: One example model between BSA and trimer of C-phycocyanin; Figure S5: One example model between BSA and hexamer of C-phycocyanin.

## Abbreviations

BSA	Bovine serum albumin
CD	Circular dichroism
ThT	Thioflavine T
