# Supplementary material for: Molecular Interaction of Protein-Pigment C-Phycocyanin with Bovine Serum Albumin in a Gomphosis Structure Inhibiting Amyloid Formation
Source: Int J Mol Sci. 2020 Nov 2;21(21):8207. doi: 10.3390/ijms21218207 (PMC7663302; doi:10.3390/ijms21218207)
Supplement: Supplementary file 1 [file ijms-21-08207-s001.pdf]

Molecular interaction of protein-pigment C-phycocyanin with bovine serum albumin in a gomphosis structure inhibiting amyloid formation

Yi-Cong Luo and Pu Jing *

Shanghai Food Safety and Engineering Technology Research Center, Bor S. Luh Food Safety Research Center, Key Lab of Urban Agriculture (South), School of Agriculture & Biology, Shanghai Jiao Tong University, Shanghai 200240, China

Contents

1.Representative bovine serum albumin CD spectra……………………..………………2
2.Detail about molecular docking…………………….……………………..…...3

1. Representative bovine serum albumin CD spectra

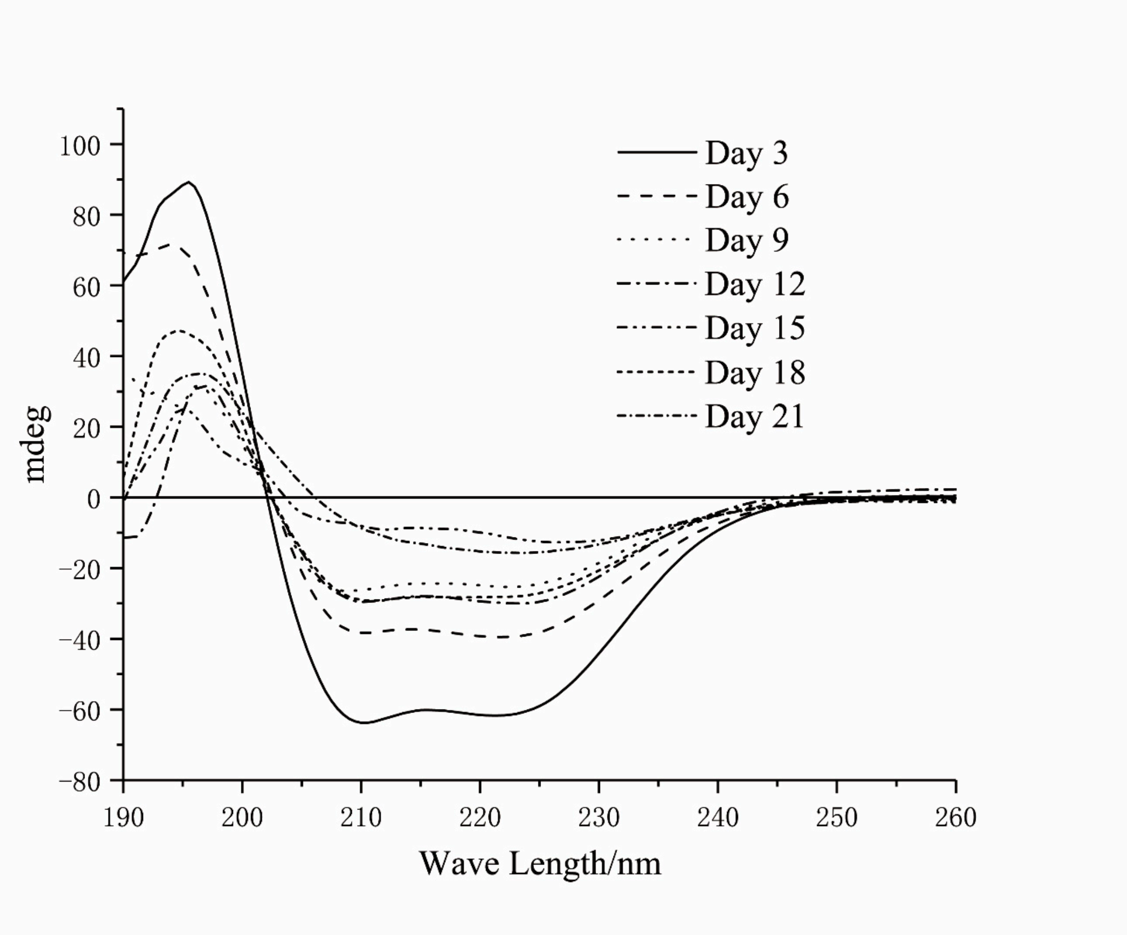

Figure S1. CD data of BSA without the existence of C-phycocyanin.

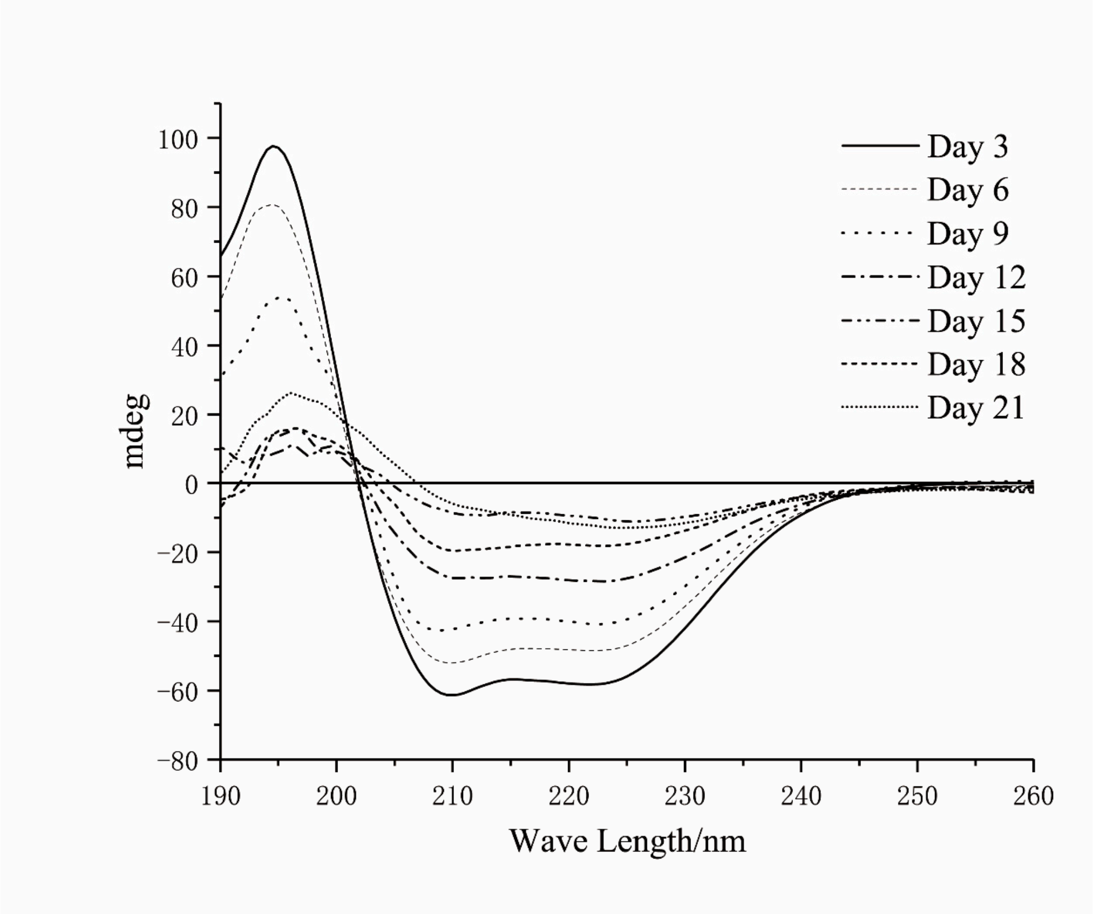

Figure S2. CD data of BSA with the existence of C-phycocyanin.

2. Detail about molecular docking

2.1. Two abandoned models between BSA and monomer of C-phycocyanin

This section provided two models between BSA and monomer of C-phycocyanin that are not selected as our main model due to less interactions and worse combining models.

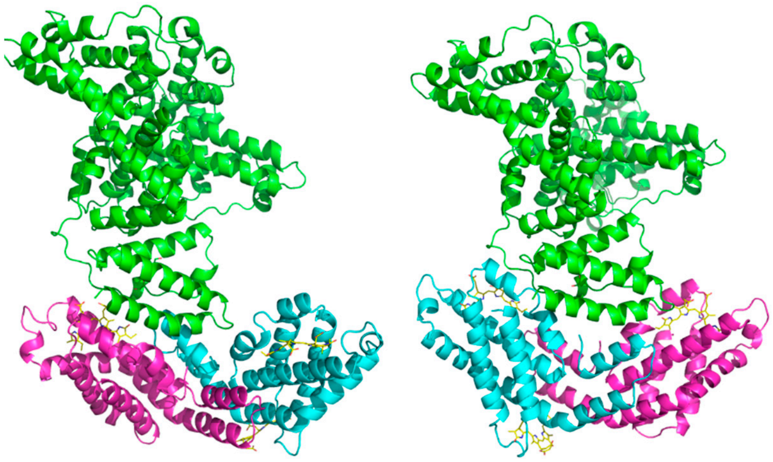

Figure S3. Two models between BSA and monomer of C-phycocyanin with less interactions.

2.2. One example model between BSA and trimer or hexamer of C-phycocyanin

Molecular model between BSA and trimer or hexamer of C-phycocyanin seemed impossible. Not only because our system was very dilute (less than 1mg/mL), but the molecular docking results suggested that compared with monomer, trimer, and hexamer of C-phycocyanin interacted much worse with BSA. The main U-shaped cavity was hidden by other C-phycocyanin and thus BSA failed to attach C-phycocyanin more tightly. In fact, trimer and hexamer of C-phycocyanin possessed a small hole that BSA was too large to enter in, yet some other small molecules or protein may fit this hole.

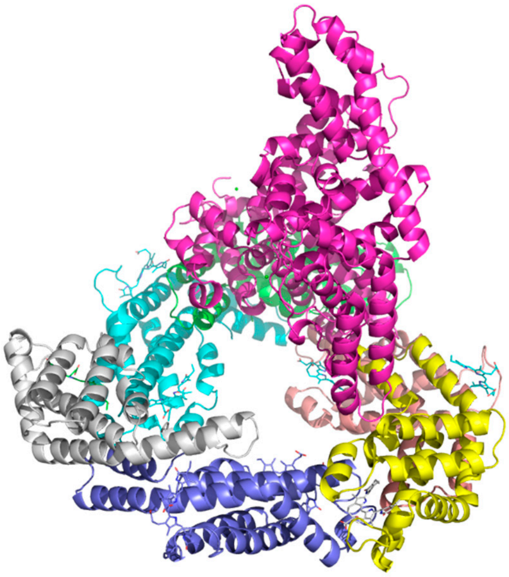

Figure S4. One example model between BSA and trimer of C-phycocyanin.

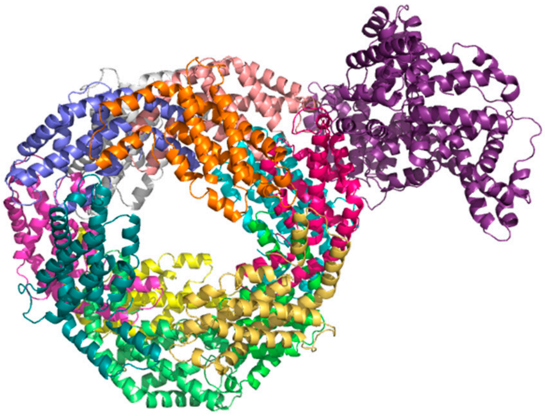

Figure S5. One example model between BSA and hexamer of C-phycocyanin.
